# Importance of sigma factor mutations in increased triclosan resistance in *Salmonella* Typhimurium

**DOI:** 10.1186/s12866-015-0444-2

**Published:** 2015-05-19

**Authors:** Mette Rørbæk Gantzhorn, John Elmerdahl Olsen, Line Elnif Thomsen

**Affiliations:** Department of Veterinary Disease Biology, University of Copenhagen, Faculty of Health and Medical Sciences, Stigboejlen 4, 1870 Frederiksberg C, Denmark

**Keywords:** Triclosan-resistance, Biocides, Antibiotic resistance, *fabI*, Efflux, Sigma-factors

## Abstract

**Background:**

*Salmonella enterica* is the second most common foodborne pathogen. The use of biocides is crucial to prevent spread of foodborne pathogens, and it would be devastating for food safety if *Salmonella* would become resistant to the disinfectants used. Another concern is that exposure to disinfectants might lead to decreased susceptibility to antibiotics.

The current study aimed to identify genetic changes causing high level triclosan resistance in *S. enterica* serovar Typhimurium and evaluate how these affected antibiotic resistance and efflux pump activity.

**Results:**

Wild type strains *S.* Typhimurium 4/74 and DTU3 were adapted to increasing concentrations of the biocide triclosan by serial passage. High level triclosan resistant isolates (MIC > 1000 μg/ml) were obtained. Strains were genome sequenced, and SNPs in *fabI, rpoS* and *rpoD* were found to be associated with high level resistance. However, work with defined mutants revealed that a SNP in *fabI* was not sufficient to obtain high level resistance. This required additional mutations in the sigma factors *rpoS* or *rpoD*. The adapted strains showed triclosan-dependent increased efflux, increased *fabI* expression and reduced susceptibility towards the antibiotics enrofloxacin and sulphamethoxazole/trimethoprim.

**Conclusions:**

Medium level triclosan resistance could be obtained by *fabI* mutations in *S.* Typhimurium, however, high level resistance was found to require sigma factor mutations in addition to a *fabI* mutation. Reduced antibiotic sensitivity was observed for the adapted strains, which could be associated with increased efflux.

## Background

*Salmonella* continues to be an important foodborne pathogen with 91.000 confirmed human cases reported in Europe in 2012 [1]. Among the serovars involved, *Salmonella enterica* serovar Typhimurium (*S.* Typhimurium) was the second most important serovar representing 22% of all confirmed cases [1]. Levels of resistance towards clinically important antibiotics, such as ciprofloxacin, are high in isolates of this serovar from animals and food [2].

Biocides are broadly used to prevent microbial growth and play an important role in preventing the spread of pathogenic bacteria. In recent years there has been increasing concern that use of biocides can select for antibiotic cross resistance [3], in addition to causing increased tolerance towards the biocides themselves. During the last 30 years several cases of bacteria developing resistance or tolerance towards biocides, and in some cases cross-resistance to antibiotics, have been reported [4]. Recently, it has been shown that “in-use” concentrations of disinfectants can select multidrug resistant mutants of *S*. Typhimurium [5].

Biocides are used in a wide variety of applications, spanning from preservatives in household-products like vacuum cleaners to disinfectants, make-up and industrial cleaning agents [6]. Especially triclosan, a chlorophenol, has been used in many products, including toothpaste, soaps and as antibacterial agent in toys, fabrics and cutting boards. The effect of triclosan has been extensively studied. It has been shown that triclosan inhibits the enoyl-acyl carrier protein reductase (FabI) which is involved in fatty-acid biosynthesis in *Escherichia coli* [7]. In addition, low concentrations of triclosan interfere with nutrient-uptake, whereas high concentrations facilitates membrane leakage by incorporation into the bacterial membrane [8].

Efflux pumps play a role in exporting toxic compounds from the cell and could be a common mechanism for antibiotic and biocide resistance [9]. The efflux systems EmrAB/AcrEF have been found to play a role in the susceptibility of *Salmonella* towards triclosan [10]. Furthermore, inactivation of the efflux pump genes *acrB* and *tolC* in *Salmonella* has previously been shown to decrease triclosan resistance [11]. However, proteomic studies of various triclosan-resistant strains revealed that there was no significant overexpression of the AcrAB-TolC efflux-pump [12], indicating that efflux is not the main mechanism of triclosan resistance [13].

Different genes have been associated with reduced susceptibility to triclosan. Results from *E. coli* and *S. aureus* have implied that point mutations in *fabI* are the primary reason for decreased triclosan sensitivity and that *fabI* overexpression in *E. coli* is associated with triclosan resistance [14, 15]. In accordance with this, up-regulation of *fabI* in *Salmonella* has been described in response to triclosan exposure, but point mutations or overexpression of *fabI* is not sufficient to give high-level resistance in this bacterium, indicating that there are other, yet to be determined, factors involved [11, 16]. The aim of this study was to determine which mutations, in addition to mutation in *fabI*, are required for *S.* Typhimurium to obtain high level biocide resistance. To study this, we adapted two different isolates of *S.* Typhimurium to high level triclosan resistance and identified the genes involved in this resistance. In addition, we investigated the effect of the mutations on efflux activity, antibiotic cross resistance and cell culture invasiveness and growth.

## Methods

### Bacterial strains

Bacteria used in this study are listed in Table 1. To evaluate the effect of single SNPs (compared to wild type) from strains adapted to high level triclosan, a single SNP was isolated from other SNPs by transfer to a clean parent strain background by phage transduction using P22 phages. P22 transductions were performed with P22HT105/int^−^201 as described [17]. A selective pressure of 2 μg/ml of triclosan was used to select for the transfer of the *fabI* SNP. An *rpoS* deletion-mutant was constructed by Lambda-Red mediated allelic exchange of the *rpoS* gene with the chloramphenicol cassette as previously described [18] using the primers rpoSfwd: 5′CAGAATACGCTGAAAGTTCATGATTTAAATGAAGACGCgtgtaggctggagctgcttc3′ and rpoSrev: 5′GCGGAACAGCGCTTCGATATTCAGCCCCTGCGTCTGCAcatatgaatatcctccttag3′ in 4/74. The mutant was verified using a PCR-strategy with the primers rpoScon: 5′-GGATCACGGGTAGGAGCCACCTTTTGAG and camfwdny: 5′-TACGCAAGGCGACAAGGTGCTGATGCCG as previously described [19]. The deletion of *rpoS* was transferred to TDTU3C by P22 phage mediated general transduction. Mutations were confirmed by PCR and sequencing. Strains were maintained in LB-Lennox broth (LB) (BD Difco™). For growth on solid media, LB was added 1.5% agar producing LB agar plates.

**Table 1:** Strains of S. Typhimurium used in this study

Strain	Properties	Origin/reference
4/74	Wild type	30
K4/74	Control, 4/74 sub-cultured without triclosan as many times as adapted strains	This study
T4/74A	4/74 adapted to triclosan	This study
T4/74B	4/74 adapted to triclosan	This study
T4/74C	4/74 adapted to triclosan	This study
4/74Δ*rpoS*:Cam	*rpoS* deletion mutant	This study
T4/74-*fabI*	*fabI* G93S	This study
DTU3	Wild type (DFVF/FOOD: 2009-60-277)	This study
KDTU3	Control, DTU3 sub-cultured without triclosan as many times as adapted strains	This study
TDTU3A	DTU3 adapted to triclosan	This study
TDTU3B	DTU3 adapted to triclosan	This study
TDTU3C	DTU3 adapted to triclosan	This study
DTU3-*fabI*	*fabI* G93S	This study
DTU3-*fabI*-*rpoS*	*rpoS* (R100H) from TDTU3C transduced into DTU3-*fabI*	This study
TDTU3CΔ*rpoS*:Cam	*rpoS* deletion mutant	This study

### Biocide solutions

A stock solution of 50 g/L triclosan (Irgasan, Sigma-Aldrich, Broendby Denmark) was made in 70% ethanol and from here a solution of 300 μg/ml was prepared in sterile MilliQ-water and kept at 5 °C until precipitations had dissolved, usually 18–20 h.

### Adaptation to high concentrations of biocides

Adaptation to high concentrations of biocides was obtained by daily sub-cultivations using an oblique-plate assay modified from assays previously described [20, 21]. Briefly, 3x700μl MHB (Müller Hinton broth, Oxoid A/S, Roskilde, Denmark) was inoculated with one colony and incubated at 37 °C with shaking for 3.5 h. Plates containing a maximum concentration of ½ MIC were streak-inoculated starting from the highest concentration in triplicate. After incubation overnight at 37 °C colony material swabbed from growth at the highest concentration were suspended in 1 ml sterile 0.9% NaCl and sub-cultivated daily on the same concentration until growth occurred in the full length of the plate on two subsequent days, then the concentration was doubled. Adaptation was ended after five subsequent days using same concentration with no increase in the extent of growth. Control-adaptations were done on plates not containing biocide to examine the effect of sub-cultivations alone. Adaptation stability was examined by growth in media without biocide for five subsequent days with daily passage.

### MIC-determinations

MIC determinations for biocides were done by micro dilution assay as previously described [22]. Strains adapted to biocides were tested in concentrations ranging from 8–4000 μg/ml and non-adapted strains (wild type and control-adapted strains) were tested in concentrations 0.14-64 μg/ml. All MIC determinations were done in technical duplicate and biological triplicate.

### Antibiotic susceptibility tests

Susceptibility towards antibiotics was examined in biological triplicate using disk diffusion according to EUCAST guidelines version 3.0, 24 April, 2013 using Müller Hinton agar (MHA) (Oxoid A/S, Roskilde, Denmark). Furthermore antibiotic resistance tests were performed on MHA containing triclosan in concentrations of 1 and 1000 μg/ml. Statistical analysis was performed on zone diameters varying >2 mm compared to wild type.

### MBC determinations

Minimum bactericidal concentrations (MBCs) towards biocide were estimated by spot plating 2x10μl on LB agar from inoculum and all wells without growth from the MIC-plate after incubation. MBC was determined as the lowest concentration giving more than a 5-log reduction of inoculum.

### Genome sequencing and SNP detection

Both parent strains and all adapted strains including controls were sequenced. DNA was extracted from overnight cultures using DNeasy Blood and Tissue Kit (Qiagen) according to manufactures instructions. Genome sequencing was done at the National High-Throughput Sequencing Centre, University of Copenhagen, Denmark using Illumina HiSeq 2000 paired end reads with a coverage of 100–200 bp. Sequences were analyzed for the presence of SNPs and other genomic variations using CLC Genomic Workbench 5.5.1 probalistic variant detection after mapping to the annotated genome of *S.* Typhimurium strain 4/74 (CP002487) [23]. Variants of DTU3 were filtered against control-reads of DTU3wt with a control read count threshold of 20 prior to analysis. Mapping of 4/74 revealed 100% coverage and the mapping of DTU3 showed 98% coverage meaning that the sequenced obtained from DTU3 covered 98% of the published genome of 4/74, which primarily contained genes encoding phage proteins, prophages and other phage related structures.

### Growth curves

Growth was evaluated with and without triclosan in a concentration of 0.3 μg/ml. Growth curves were obtained using Bioscreen C (Finland) in a final volume of 200 μl Müller Hinton broth (MHB). The inoculum was 5 μl overnight (ON) culture diluted to an OD_600_ = 0.05, giving a final OD in the wells of 0,001 (about 1*10^6^ CFU/ml). Plates were incubated at 37 °C in Bioscreen C with medium shaking for 10 sec before each measurement. OD_600_ was measured every 20 min for 23 h. Growth curves were performed in biological triplicate.

### Efflux pump activity

Efflux pump activity was evaluated using the Cartwheel method modified from [24]. The inoculum was prepared by growing the strains for 4 h in LB at 37 °C with shaking. Efflux was evaluated in increasing concentrations of triclosan (0.25–1000 μg/ml) with fixed concentration of ethidium bromide of 1 μg/ml. Fluorescence was examined using GelDoc (Bio-Rad Laboratories, Inc.).

### Northern blot

Northern blot analysis was performed as previously described [25]. Cells at mid-exponential phase (OD_600_ = 0.5+/-0.05) and stationary phase (OD_600_ = 1.2 +/−0.1) grown with and with-out triclosan at a concentration of 0.3 μg/ml were harvested by centrifugation for 8 min at 10.000 rpm. The pellet was stored at −80 °C. Probes targeting *fabI* transcript was amplified by PCR using fabIf: 5′AAGCGCATTCTGGTCACTGG3′ and fabIr: 5′TTCAATGGTCACGGTGCGAC3′ and labeled with [α-^32^P]dCTP.

### Cell culture infections

Cell-assays were performed using INT-407 (HeLa contaminated epithelial cell-line) as previously described [26] with few modifications: Cells were seeded at 5x10^5^ cells/well in 24-well plates and grown overnight at 37 °C supplemented with 5% CO_2_ in MEM (1X) + GlutaMAX™ –I (Gibco ® by life technologies ™) supplemented with 10% heat treated Fetal Bovine Serum (FBS) (Gibco ® by life technologies ™). Bacteria from overnight LB cultures were diluted to OD_600_ = 0.05 and grown for two hours before harvesting and adjusting OD_600_ to 0.1. Cells were infected in technical duplicate with a MOI of 50:1 for 30 min before wash with warm 0.9% NaCl. For determination of invasion wells were added 0.5 ml fresh MEM + 10% FBS with 100γ Gentamycin and incubated for 2 h at 37 °C supplemented with 5% CO_2_. At both time-points (30 min and 30 min + 2 h) cells were washed and lysed using 0.1% TritonX-100 before serial dilution and plating at LB agar for CFU counts. Lysates and inoculum was plated on LB agar in technical duplicate to establish CFU/ml and hence calculate adhesion-and invasion-efficiency. Cell-assays were done in biological duplicate.

### Statistics

For the analysis of differences in antibiotic resistance a mixed linear model was used. Each concentration of triclosan was analyzed separately, and zone diameters of the adapted strains at triclosan concentration of 1000 μg/ml were compared to wild type strains at triclosan concentration of 1 μg/ml. Zone diameter for each antibiotic were the response variable and strain were the explanatory variable. Date of antibiotic testing was included as a random factor. The statistical analysis was performed using the program R [27] and the packages “lme4” [28] and “multcomp” [29]. Student’s t-test in Excel 2010 (Microsoft) was used to analyse differences in adhesion and invasion between strains. Significance levels were set at 5%.

## Results

### Salmonella Typhimurium isolates with tolerance to high concentration of triclosan are easily obtained after exposure to triclosan

Two different *S.* Typhimurium strains, the clinical isolate 4/74 [30] and the pork slaughterhouse isolate DTU3 were exposed to triclosan to evaluate the ability to obtain resistant mutants and to elucidate the genetics behind this adaptation. Adaptation was done using daily sub-culturing on gradient plates containing increasing concentrations of triclosan. Three biologically independent adapted strains were isolated for each wild type strain. Before adaptation, the triclosan MIC values for 4/74 and DTU3 were 1 μg/ml and 8 μg/ml, respectively. After 36 sub-cultivations the MIC had increased to 1057–2088 μg/ml for strain 4/74, subcultures A-C (from here on designated T4/74 strain A-C) and 2088–4124 μg/ml for TDTU3 strains A-C (Table 2). MBC-values, defined as the lowest concentration giving a 5-log reduction of inoculum, were shown to be 4124 μg/ml and 4124–8146 μg/ml respectively (Table 2). To evaluate the stability of the phenotype, the adapted strains were subsequently grown five days without triclosan and were found to maintain their high level resistance (data not shown). This indicated that the adaptation was caused by genetically inheritable mutations rather than development of phenotypic tolerance. The control strains K4/74 and KDTU3 were similarly exposed to 36 sub-cultivations, but without triclosan. Both control strains maintained the wild type MIC (Table 2).

**Table 2:** MIC and MBC values (μg/ml) of triclosan for *S* Typhimurium wild type and mutants

Strain	MIC	MBC
4/74 (wild type)	1	>64
K4/74	0.5	ND
T4/74A	2088	4124
T4/74B	1057	4124
T4/74C	2088	4124
4/74-*fabI*	270	ND
DTU3 (wild type)	8	>64
KDTU3	8	ND
TDTU3A	2088	4124
TDTU3B	2088	8146
TDTU3C	4124	8146
DTU3-*fabI*	64	ND
DTU3-*fabI-rpoS*	4124	ND
TDTU3CΔ*rpoS*::Cam	≤0.14	ND

ND: Not determined

### Concurrent mutations in *fabI* and sigma-factor leads to high-level resistance

To elucidate the changes responsible for triclosan resistance, genome sequencing of the strains was conducted. The genome sequences of the adapted strains were compared to th**e** corresponding control strains and revealed between two to seven changes (Table 3). Only non-synonymous changes in coding regions were considered. A Gly-93 → Ser mutation in *fabI* was present in all adapted strains with the exception of TDTU3C for which a Gly-93 → Val mutation in *fabI* was present. Mutations were also found in several other genes including *rpoD* and *ndh* (T4/74C) and *rpoS* (TDTU3C) (Table 3)*.* We focused our investigations on the two adapted strains, T4/74C and TDTU3C to elucidate the importance of the SNPs in the sigma factors, *rpoD* and *rpoS*. To investigate whether mutations in the adapted strains had an effect on fitness, their growth was compared to the control strains. We found that the adapted strains T4/74C and TDTU3C and their corresponding control strains showed similar growth (data not shown). To evaluate the importance of the sigma factor SNPs, we constructed a mutant harboring only the *fabI* mutation by transferring the *fabI* SNP of T4/74C to the wild type strain 4/74, obtaining the strain 4/74-*fabI*. This strain had higher MIC (270 μg/ml) than parent wild type strain, but did not show the same high MIC to triclosan as the adapted strain, that also contained the *ndh* and *rpoD* mutations (Table 2). Similarly, the MIC of a DTU3-*fabI* mutant (64 μg/ml), without the SNP in *rpoS* was also found to be 4 times higher than the wild type, but 64 fold lower than the TDTU3C strain (Table 2). Introducing the *rpoS* mutation (R100H) into DTU3-*fabI* by phage transduction re-established the high triclosan MIC, confirming that both mutations have to present to obtain high level resistance (Table 2). The importance of a mutation in a sigma factor was consistent with the observations in the adapted strain TDTU3C, which only had mutations in the sigma factor *rpoS* in addition to the mutation in *fabI.* Furthermore, the significance of a functional sigma factor for high triclosan resistance was shown by deleting the *rpoS* gene in the TDTU3C strain resulting in the mutant TDTU3C∆*rpoS*::cam which had lost its high level resistance (Table 2).

**Table 3:** Non-synonymous mutations within coding regions of adapted strains

Protein	T4/74A	T4/74B	T4/74C	TDTU3A	TDTU3B	TDTU3C
STM474_0600 Putative regulatory protein		Q19*				
Ndh NADH dehydrogenase		G66V	G66V			
SelD Selenophosphate synthetase	D327V					
STM474_1682 Invasion-like protein	F131fs					
FabI enoyl-(acyl-carrier protein) reductase	G93S	G93S	G93S	G93S	G93S	G93V
RfbV Abequosyl transferase	K177fs					
RcsB Transcriptional regulator	V149fs					
RpoD RNA polymerase sigma factor			T119I			
STM474_2675 Putative phosphotransferase system IIB component	A83V					
Fis DNA binding protein	A78D					
STM474_0304 Putative RHS-like protein				F25V	F25V	
NadE NAD synthetase				L214S	L214S	
RpoS RNA polymerase sigma factor						R100H
SspA Stringent starvation protein A				E153*	E153*	
TrpS Tryptophanyl-tRNA synthetase				T78I	T78I	

*: stop codon, fs: frame shift-leading to stop codon

Mutations in *fabI* was found in all our adapted strains and increased expression of *fabI* has previously been shown to affect triclosan resistance [11]. To evaluate if the SNPs identified altered the expression of *fabI*, the amount of *fabI* mRNA was compared between the adapted strains and the wild type. Examination of *fabI* expression using Northern Blot showed a minor up-regulation of *fabI* in the two adapted strains (data not shown). This corresponds with previously published observations of triclosan-induced *fabI* expression [11, 31].

### Antibiotic resistance and efflux pump activity in triclosan adapted strains

There is an increasing concern that adaptation to biocides could lead to cross resistance to other antimicrobial compounds, including antibiotics used in the clinical setting [6]. Antibiotic resistance analysis was done using disc-diffusion on plates with and without triclosan. When comparing wild type, control and adapted strains, we observed only slight decreases in zone diameter for the adapted strain T4/74C with enrofloxacin (ENR), and for TDTU3C exposed to sulfamethoxazole/trimethoprim (SXT),, indicating reduced sensitivity (Table 4). On the other hand, the adapted strain T4/74C showed a small increase in zone diameter regarding amoxicillin/clavulanic acid (AMC) and TDTU3C regarding ENR. However, when the tests were done using plates with triclosan in concentrations of 1000 μg/ml a highly significant decrease in zone-diameter was seen towards SXT (p < 0.001) and ENR (p < 0.001) for both T4/74C and TDTU3C (Table 4). The opposite was observed regarding cefotaxime (CTX), where both of the adapted strains showed a significant increase in zone diameter, and similarly for 4/74C exposed to ceftiofur (EFT) (p < 0.001).

**Table 4:** Zone diameters of wild type and adapted strains without and with concurrent exposure to triclosan

	Triclosan concentration (μg/ml)	TET	SD	SXT	SD		ENR	SD		AMC	SD		GEN	SD	EFT	SD		AMP	SD		CTX	SD	
4/74	0	24	2,04	25	1,47		32	2,40		24	3,39		25	3,35	26	2,35		23	4,29		32	3,58	
K4/74	0	24	1,14	26	1,58		33	1,95		24	3,49		24	4,03	25	0,71		23	2,68		33	0,96	
T4/74C	0	23	1,47	25	1,05		30	3,29		27	2,17	*	23	2,41	26	1,63		24	2,58		34	1,67	
4/74	1	25	1,41	26	0,00		35	1,41		25	4,24		29	7,07	28	3,54		25	7,78		37	3,54	
K4/74	1	25	0,71	27	2,12		34	0,71		25	2,12		27	2,83	26	0,71		25	1,41		34	0,71	
T4/74C	1	24	1,76	24	1,37		30	3,20		26	1,83		24	2,86	25	2,48		24	2,28		33	2,83	
T4/74C	1000	24	5,18	14	1,17	***	11	1,75	***	32	6,57		29	6,00	33	1,67	***	31	8,35	nd	43	3,83	***
DTU3	0	6	0,00	22	2,06		29	1,15		18	4,43		26	1,53	24	1,71		6	0,00		30	1,53	
KDTU3	0	6	0,00	21	1,22		29	1,22		17	4,69		25	0,96	24	1,48		6	0,00		30	1,00	
TDTU3C	0	6	0,00	20	1,67		30	1,10		17	4,34		24	3,10	24	1,52		6	0,00		31	0,82	
DTU3	1	6	0,00	22	1,41		31	0,71		17	4,95		27	1,41	25	0,71		6	0,00		31	2,12	
KDTU3	1	6	0,00	23	0,71		31	0,71		15	3,54		26	0,71	25	0,71		6	0,00		31	3,54	
TDTU3C	1	6	0,00	21	1,52		29	0,89		16	4,51		26	1,26	25	1,52		6	0,00		31	1,00	
TDTU3C	1000	6	0,00	13	1,79	***	11	0,84	***	19	5,18		27	2,75	26	1,14		6	0,00		35	3,79	*

TET: Tetracycline, SXT: trimethoprim/sulfamethoxazole, ENR: enrofloxacin,AMC: ampicillin/clavulanic acid, GEN: gentamicin, EFT: ceftiofur, AMP: ampicillin, CTX: Cefotaxime. Susceptibility towards antibiotics was examined in biological triplicate. Asterisk indicate level of significant difference from wild type (4/74 or DTU3): *p ≤ 0.05, ***p ≤ 0.001, nd: not determined

**Fig. 1:**
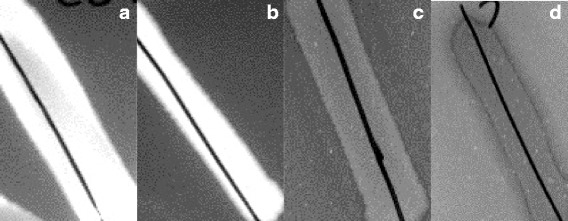
Fluorescence decreases at high concentrations of triclosan indicating higher efflux. High level of fluorescence of TDTU3C is observed at triclosan concentrations of 0,5 μg/ml (**a**) and 5 μg/ml (**b**) indicating low efflux of EtBr. Low fluorescence is seen at triclosan concentrations of 500 μg/ml (**c**) and 1000 μg/ml (**d**) indicating high efflux. Concentration of ethidium bromide is 1 μg/ml

Triclosan resistance can be caused by efflux pumps which are also known to affect resistance to antibiotics [32]. Since we observed antibiotic cross resistance when high triclosan concentrations were present, we investigated whether increasing triclosan concentrations could lead to increased efflux. We evaluated efflux activity by using accumulation of ethidium bromide (EtBr) as an indicator of the level of efflux. EtBr traverses the bacterial cell wall and once inside it binds to DNA and fluoresces in ultraviolet light. Bacterial efflux pumps recognize EtBr and are able to extrude it to the medium meaning that low fluorescence is indicative of high efflux. Our experiments revealed a correlation between efflux and concentration, showing increased efflux when the adapted strain was exposed to triclosan in high concentrations (Fig. 1). At low concentrations (0,5 and 5 μg/ml), high level fluorescence was observed as a highly fluorescent streak of bacteria. This corresponds with high level of intracellular EtBr caused by low efflux. At 500 and 1000 μg/ml triclosan, the bacterial streak only shows low level fluorescence, which indicates high efflux and limited binding of EtBr to the DNA in the cell.

### Cell culture adhesion and invasion of triclosan adapted strains

**Fig. 2:**
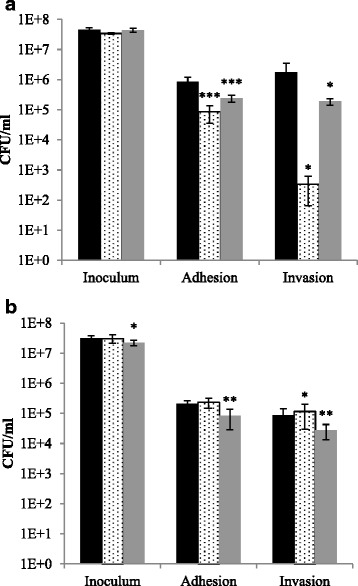
Ability of the adapted strains to adhere to and invade the INT-407 cell line. (**a**) 4/74, (**b**) DTU3, wild type (black bar), control (dotted bar) and adapted strain (grey bar). Bars represent CFU in inoculum, of adhered bacteria and invaded bacteria. Error bars represent one standard deviation. Asterisk indicate level of significant difference from wild type: *p ≤ 0,05, **p ≤ 0,01, ***p ≤ 0,001

Previous results have shown a link between triclosan resistance and virulence factor expression in Gram positive bacteria and reduced invasiveness after prolonged treatment of *S.* Typhimurium with disinfectants [4, 20]. We therefore investigated the ability of the adapted strains to adhere to and invade the cell-line INT407. We found a small, but significantly decreased ability of the adapted strain TDTU3C to adhere and invade compared to the wild type and control strains. However, the inoculum was also slightly decreased for the TDTU3C strain. 4/74C did not show reduced adherence and invasion compared to the control strain K4/74 (Fig. 2). The control strain K4/74 revealed a decreased ability to invade compared to the wild type, however it also showed a decreased adhesion, which could account for the lower ability to invade. Our results therefore indicate that the mutations do not have any major effect on virulence for the two strains.

## Discussion

In the present study, we have investigated the genetic background for the ability of *S.* Typhimurium to adapt to triclosan. Adaptation was found to be relatively easy and we were able to adapt two different strains of clinical and slaughterhouse origin to grow in concentrations equal to or above 1000 μg/ml.

The mechanisms of resistance towards triclosan have been studied quite intensively, but still the exact mechanisms are not fully understood [11–13, 16, 33–36]. To further elucidate the genetics behind this adaptation, we genome sequenced the adapted strains and compared the sequence to the control strains. In all adapted strains, a mutation was present in *fabI*, however other mutations were also present. From our genome sequencing results and subsequent construction of mutants containing *fabI* from the adapted strains, we can conclude that having a mutation in *fabI* leads to increased triclosan tolerance, but is not enough to confer high level resistance.

Our results show that mutations in one of the sigma factors *rpoS* or *rpoD,* in addition to the *fabI* mutation, caused high resistance levels in T47/4C and TDTU3C. This corresponds with previous results showing that *fabI* mutations are involved in triclosan resistance, but do not alone account for high level resistance [11]. The authors demonstrated that a variety of other genes, including *arcB*, *tolC* and *ramA* can be involved in triclosan resistance [11]. Although, it has been shown, that triclosan induce *rpoS* expression in *S.* Typhimurium [16], implying its importance for a triclosan response, we show, to the best of our knowledge, for the first time that mutations in sigma factors contribute to triclosan resistance. Sigma factors are required for transcription initiation and mutations in *rpoS* or *rpoD* indicate that changes in gene expression levels influence high level triclosan resistance. Our data show a triclosan dependent increase in *fabI* expression and increased efflux pump activity, both of which could be the result of changed expression levels caused by the sigma factor mutations.

In addition to the *fabI* and sigma factor mutation, we also found a mutation in *ndh* in T4/74C. The function of *ndh* is not fully understood, but it has been proposed that it is involved in rapid NADH recycling [37]. Since triclosan is known to bind NADH, it is not unlikely that this mutation has some additional effect on susceptibility or fitness of the strain. However, since we have found no mutations in *ndh* or other similar genes in TDTU3C, we did not consider the *ndh* mutation to be as important as the mutation in the sigma factor. It is, however, important to notice, that high level resistance to triclosan can be achieved with no mutations in sigma factors or *ndh*, as evident from the adapted strains T4/74A, TDTU3A, and TDTU3B. Further studies are needed to elucidate whether such strains harbours mutations in genes that are directly or indirectly regulated by RpoS or RpoD, eliminating the need for mutation in the regulatory gene(s).

Several researchers have been able to adapt *Salmonella* and other bacteria to certain biocides and the concern is that the mechanisms resulting in biocide resistance may confer cross resistance to antibiotics [4, 38, 39]. In this study, we found a decreased susceptibility of T4/74C and TDTU3C towards enrofloxacin and sulfamethoxazole/trimethoprim, respectively.

A highly interesting observation was that concurrent exposure to high concentrations of triclosan conferred a marked decrease in zone diameters of the adapted strains towards enrofloxacin and sulfamethoxazole/trimethoprim. A possible explanation could be the induction of efflux by triclosan. We found that increased efflux of EtBr was linked to increased concentration of triclosan (Fig. 1) supporting the hypothesis that high triclosan concentrations induce efflux and thereby explain why cross resistance to antibiotics is primarily observed when the bacteria are also subjected to high concentrations of triclosan. This is likely since resistance towards SXT and fluoroquinolones can occur via efflux. The ArcAB-TolC efflux pumps are important for triclosan resistance [11] and these pumps have also been shown to directly contribute to flouroquinolone (e.g. ENR) resistance in *Salmonella* [40, 41]. The efflux systems EmrAB and ArcEF have been shown to be important for both triclosan resistance [10] and trimethoprim resistance [42]. Our results support the previous observations of triclosan dependent increase of antibiotic resistance in different bacteria [43–46].

Our data demonstrated that the mutations in T4/74C only had minor effects on adhesion and invasion to a cultured cell line. In contrast to this, the mutations in TDTU3C did have significant effect on adhesion and invasion, however, the inoculum was slightly lower for this strain compared to the wild type strain, and this might explain this difference. Previous work on triclosan exposed *S.* Typhimurium has showed reduced ability to invade Caco-2 epithelial cells [4]. This difference could be due to differences in cell lines and different mutations in the strains investigated.

## Conclusion

The current study has demonstrated that high level resistance to triclosan can be achieved by mutations in both *fabI* and in a sigma factor. The combinations of these mutations do not compromise growth, but induces efflux, which in turn may be accountable for the observed antibiotic resistance. Further research is needed to clarify the role of sigma factor mutations in high level triclosan resistance.

## Abbreviations

CFU: Colony forming units
